# Scaffold filling, contig fusion and comparative gene order inference

**DOI:** 10.1186/1471-2105-11-304

**Published:** 2010-06-04

**Authors:** Adriana Muñoz, Chunfang Zheng, Qian Zhu, Victor A Albert, Steve Rounsley, David Sankoff

**Affiliations:** 1 School of Information Technology and Engineering, University of Ottawa, Ottawa, K1N 6N5, Canada; 2 Département d'informatique et de recherche opérationnelle, Université de Montréal, Montréal, H3C 3J7, Canada; 3 Department of Computer Science, Princeton University, Princeton, NJ 08544, USA; 4 Department of Biological Sciences, University at Buffalo, Buffalo, NY 14260, USA; 5 School of Plant Sciences and BIO5 Institute, University of Arizona, Tucson, AZ 85719, USA; 6 Department of Mathematics and Statistics, University of Ottawa, Ottawa, K1N 6N5, Canada

## Abstract

**Background:**

There has been a trend in increasing the phylogenetic scope of genome sequencing without finishing the sequence of the genome. Increasing numbers of genomes are being published in scaffold or contig form. Rearrangement algorithms, however, including gene order-based phylogenetic tools, require whole genome data on gene order or syntenic block order. How then can we use rearrangement algorithms to compare genomes available in scaffold form only? Can the comparative evidence predict the location of unsequenced genes?

**Results:**

Our method involves optimally filling in genes missing from the scaffolds, while incorporating the augmented scaffolds directly into the rearrangement algorithms as if they were chromosomes. This is accomplished by an exact, polynomial-time algorithm. We then correct for the number of extra fusion/fission operations required to make scaffolds comparable to full assemblies. We model the relationship between the ratio of missing genes actually absent from the genome versus merely unsequenced ones, on one hand, and the increase of genomic distance after scaffold filling, on the other. We estimate the parameters of this model through simulations and by comparing the angiosperm genomes *Ricinus communis *and *Vitis vinifera*.

**Conclusions:**

The algorithm solves the comparison of genomes with 18,300 genes, including 4500 missing from one genome, in less than a minute on a MacBook, putting virtually all genomes within range of the method.

## Background

The dramatic drop in the expense of genome sequencing has two somewhat contradictory effects on the study of gene order. On one hand it greatly increases the range of organisms available for genomic analysis, including comparative studies and phylogenomics. On the other hand, however, it encourages the final release of the genomes in unfinished *(standard *or *high-quality *draft) form, since the cost of finishing has not decreased at nearly the same rate as the cost of random sequencing [1]. The use of draft genomes makes many analyses and interpretations tentative and prone to error, and leads to particular problems in the comparative study of gene order. Many algorithms for studying genome rearrangement require whole genome data, i.e., complete representations of each chromosome in terms of gene order, conserved segment order, or some other marker order, in order to calculate the rearrangement distance *D *between two genomes. Items whose chromosomal location is unknown cannot be part of the input. This puts the many draft genomes outside the scope of currently available comparison technology, even though these data may be suitable to other goals of genomics.

### Strategy

To overcome these hindrances to the exploitation of much of the genome sequence data produced now and in the future, we have undertaken a program of adapting genome rearrangement methodology to partially sequenced and incompletely assembled genomes. The idea is to use comparative information algorithmically to improve the assembly of a draft genome, including the ordering of scaffolds on the chromosomes and the insertion of unsequenced genes in scaffold gaps, while simultaneously using the improved assemblies in comparison of gene order and inference of genome rearrangement. In earlier studies on papaya *Carica papaya *[2] and *Drosophila *[3], we investigated the case when one or both of the genomes being compared are given only in contig form. Though we did manage to find appropriate genomic data in contig form to test our methods in these studies, most sequencing projects are able to order some or all of the contigs, with intervening gaps, in scaffolds, which contain more information than unordered sets of contigs. In the next section, we model how contigs are organized into scaffolds in the two current approaches to sequencing. We then formalize scaffolded genome comparison, where one of the genomes is known only in scaffold form, as a combinatorial optimization problem for inserting missing genes in the scaffold gaps in such a way as to minimize the rearrangement distance. We devise an exact polynomial-time solution for this problem. We then assess how this algorithm performs on simulated data and apply it to compare the scaffolded genome of castor bean *Ricinus communis *to the fully sequenced genome of grapevine *Vitis vinifera*. In the process, we discover how to estimate what proportion of the missing genes are simply unsequenced or unidentified, and what proportion are actually absent from the genome.

Although using comparative evidence has long been commonplace in predicting gene location and indeed is one of the original motivations for model organism genomics, we believe this to be the first effort to predict the locations of large numbers of genes simultaneously using combinatorial optimization, while detecting and taking account of genome rearrangements.

### Partial sequencing scenarios

By *contig *we understand a completely sequenced fragment of a chromosome. This is assembled through identifying significantly overlapping reads of sequencing reactions. By *scaffold *we mean a set of ordered contigs (the order reflecting that on the chromosome) separated by unsequenced DNA which may be of known or unknown length. An anchored scaffold or contig is one whose location on the chromosome is known, thanks to any one of a number of different types of evidence.

**Figure 1:**
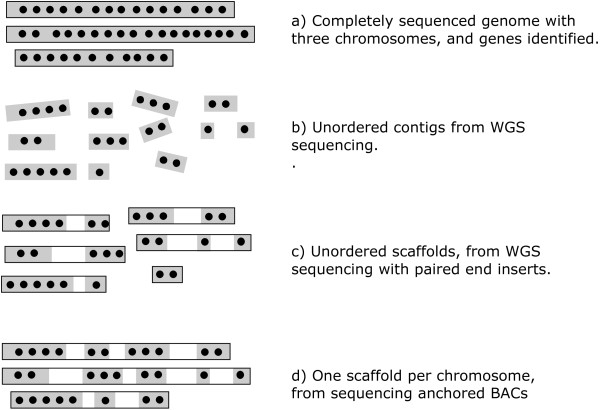
**Types of partially sequenced and incompletely assembled genomes**. Shaded areas represent sequenced contigs. Dots represent identified genes. The set of contigs within each outlined portion has a known order.

In an idealized completely sequenced and gene-identified genome, complete gene orders would be known for each chromosome (Figure 1a). When genome sequencing is not supplemented by finishing techniques, however, three different types of incomplete gene order data can result. When a strategy such as shotgun sequencing of unordered clones is employed, we have only isolated contigs constructed from overlapping reads, which would contain no internal gaps but could be relatively short assemblies (Figure 1b).

Contigs-only assemblies could also involve much longer sequence fragments produced by complete, polished, sequencing of BACs or other chromosome fragments, which are not yet numerous enough to have been assembled into full chromosomes. When *paired ends *reads with unsequenced inserts are included with shorter complete reads, some of the contigs may then be ordered into scaffolds, with unsequenced gaps intervening between successive contigs, as in Figure 1c. Finally, detailed physical maps may be available to anchor all scaffolds to precise chromosomal locations, so that the scaffolds for a given chromosome become, in effect, a single scaffold or *pseudomolecule *(Figure 1d).

In practice, sequencing projects may use both BAC and shotgun methods as well as sequence obtained by other means. Not all BACs are necessarily anchored and some contigs produced by shotgun methods may be anchored. Nevertheless, from our viewpoint, the three abstractions represented in Figure 1b), 1c) and 1d) capture the essential distinctions between contig and scaffold and between anchored and unanchored.

### Genomic distance

**Figure 2:**
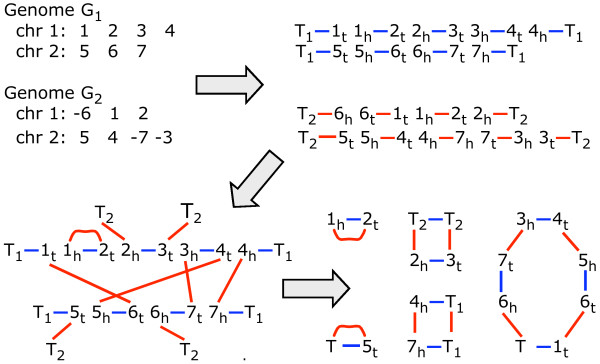
**Construction of breakpoint graph**. Upper left: Signed genomes *G*_1 _and *G*_2_. Upper right: Vertices and edges of individual genome graphs. Lower left: Cycles and paths after identifying vertices of two genome graphs. Lower right: Cycles in completed breakpoint graph.

The *rearrangement distance *or *genomic distance D*(*G*_1_, *G*_2_) is a metric counting the number of rearrangement operations necessary to transform one signed multichromosomal gene order *G*_1 _into another *G*_2_. In the simplest case, we require that the two genomes both contain the same *n *genes, with no duplicate genes. The positive or negative sign associated with a gene indicates its reading direction (or strandedness). To calculate *D *efficiently, we use the *breakpoint graph *of *G*_1 _and *G*_2 _as follows and as illustrated in Figure 2.

In a first step, each gene *g *with a positive sign is replaced by its tail and head vertices in the order *g*_*t*_, *g*_*h*_; for -*g *we would put *g*_*h*_, *g*_*t*_. Each pair of successive genes in the gene order defines an adjacency, namely the pair of vertices that are adjacent in the vertex order thus induced. For example, if *i*, *j *-*k *are three neighbouring genes on a chromosome then the unordered pairs {*i*_*h*_, *j*_*t*_} and {*j*_*h*_, *k*_*h*_} are the two adjacencies they define. There are two special vertices called telomeres for each linear chromosome, namely the first vertex from the first gene and the second vertex from the last gene.

We convert all the telomeres in genome *G*_1 _and *G*_2 _into adjacencies with new vertices all labelled *T*_1 _or *T*_2_, respectively. We define a blue edge connecting the vertices in each adjacency in *G*_1 _and a red edge for each adjacency in *G*_2 _.

In the next step in Figure 2, we start constructing the breakpoint graph by identifying (i.e., superimposing) each vertex in *G*_1 _with the identically labelled vertex in *G*_2_. In the last step depicted in Figure 2, we make a cycle of any path ending in two *T*_1 _or two *T*_2 _vertices, connecting them by a red or blue edge, respectively, while for a path ending in a *T*_1 _and a *T*_2 _, we collapse them to form one *T *vertex.

Each vertex is now incident to exactly one blue and one red edge. This bicoloured graph decomposes uniquely into *κ' *alternating cycles. If *n' *is the number of blue edges(1)

and the optimizing rearrangements are rapidly recovered by operations on the graph [4,5].

In the **Methods **section below, we will refer to Tesler's [6] mathematically equivalent formulation of the breakpoint graph, where the final step in Figure 2, turning paths into cycles, is not carried out. Instead, there are only *κ *≤ *κ' *cycles and a certain number *π *of the paths, namely those with at least one *T*_1 _endpoint, are called *good *paths. Then(2)

where *χ*_1 _is the number of chromosomes in *G*_1_. Although the breakpoint graphs, and *D*, are equivalent in the two formulations, Tesler does not call *D *"genomic distance". This difference is due to our inclusion of transpositions of chromosomal segments in the repertoire of rearrangements permitted in calculating *D*, together with the inversions, reciprocal translocations, chromosome fusions and fissions allowed by Tesler.

## Methods

There are two different aspects of the comparison of a completely assembled genome *G_1 _*with a genome in scaffold form *G*_2_. One is *scaffold filling*, which predicts where in *G*_2 _to locate potential genes that have not been identified in the sequence but are present in *G*_1_. The second is *contig fusion*, which suggests how to piece *G*_2 _contigs together to form chromosomes. In Figure 1, only scaffold filling is necessary for scenario (d) and only contig fusion is required for scenario (b). Scenario (c) requires both.

We have shown how to handle the contig fusion problem in previous publications on papaya [2] and on *Drosophila *[3], and this will be reviewed in a separate section below. In the present paper we design and analyze an efficient exact algorithm for scaffold filling that simultaneously carries out contig fusion. We use this algorithm to analyze real and simulated data.

### Filling in scaffolds

When *G*_2 _is only partially sequenced, and is missing some orthologs with *G*_1 _(cases (c) and (d) in Figure 1), we cannot complete the breakpoint graph since the red edges cannot be drawn to the two vertices corresponding to each missing gene, though these vertices are present in the graph and are incident to blue edges. At the same time, although we can draw a red edge between the last gene in one contig of a scaffold and the first gene in the next contig, we know that in reality there may be genes in the unsequenced gap between the contigs, and that once these genes are identified, the red edge will have to be "cut" and replaced by two or more gene vertices and two or more other red edges.

### Statement of the combinatorial optimization problem

*G*_1 _consists of *χ *chromosomes, each of which is an ordered set of signed genes.

A contig in *G*_2 _is an ordered set of one or more signed genes, each orthologous to a gene in *G*_1_. A scaffold in *G*_2 _is an ordered set of contigs. Then *G*_2 _consists of a number of scaffolds, each of which is an ordered set of genes interrupted occasionally by a gap.

Then with reference to Figure 1(c) and (1d), the problem becomes: Find an assignment of the missing genes to the gaps in the scaffolds or at the ends of the scaffolds of *G*_2_, thus transforming the scaffolds into contigs, such that the resulting set of contigs  is at a minimum rearrangement distance from *G*_1_.

Implicit in our definitions is that between every pair of successive contigs in a scaffold is a gap large enough to contain genes. Where this is not the case, we can simply create a larger contig by disregarding the gap and concatenating the contigs on either side. We also disregard contigs without genes, so that they too may be subsumed in a gap. Note these are basically terminological conventions, rather than restrictions on the data.

### A polynomial-time algorithm

The exact, linear-time, algorithm we have devised completes the breakpoint graph, only partially determined by *G*_1 _and by the scaffolds of *G*_2_, by means of insertions of missing genes into the gaps of *G*_2_.

#### Terminology

We have hitherto used the term *path *only to refer to alternating-colour sequences of edges connecting some of the bivalent vertices in the breakpoint graph, with telomeres at either end, that are eventually turned into cycles by joining or collapsing these two telomeres. In what follows, however, a path more generally may be any such connected set of edges, with or without telomeres, and may consist of only one (blue) edge. Paths with two telomeres will be called *complete *paths.

A *free end *is a vertex in the graph that has no incident red edges, only a blue one.

Thus when we say that that *G*_1 _and the scaffolds of *G*_2 _*partially determine *a breakpoint graph, we mean that there are paths not ending in two *T *vertices, but in at least one free end.

A *half path *is a path ending in one telomere and one free end. A *pseudopath *is a structure consisting of two half paths where the two telomeres are deemed to be adjacent, though not by means of a red or blue edge. Pseudopaths will sometimes be treated as if they were paths, with the two free ends being the free ends from the two constituent half paths.

Initially, a *cuttable edge *is a red edge drawn between vertices of two successive genes in a scaffold that are not in the same contig, i.e., there is an unsequenced gap between the genes. Subsequently, if a red edge is disrupted during gene insertion, new red edges are created as will be specified in the algorithm presented below.

A *bundle *is a subset of the paths in the breakpoint graph of *G*_1 _and *G*_2_. Each bundle is associated with one or more of the missing genes. The vertices corresponding to each missing gene, its free ends, must be in the same bundle and must be endpoints of two paths, or the two ends of one path. An *open *bundle contains at least one cuttable edge; a *closed *one has no cuttable edges. As the breakpoint graph is completed by the algorithm, the bundles also change.

#### A sketch of the algorithm

We have divided the algorithm into three parts. The first, the main algorithm **fillScaffolds**, constructs the partial breakpoint graph determined by *G*_1 _and the scaffolds of *G*_2_, and then partitions the paths in this graph (except the complete paths, and not including the cycles) among a number of bundles, some open and some closed. Initially, a bundle can contain either zero or two telomeres. If they are present, the half-paths, which are the two paths ending in telomeres, are linked together to become a pseudopath.

**Figure 3:**
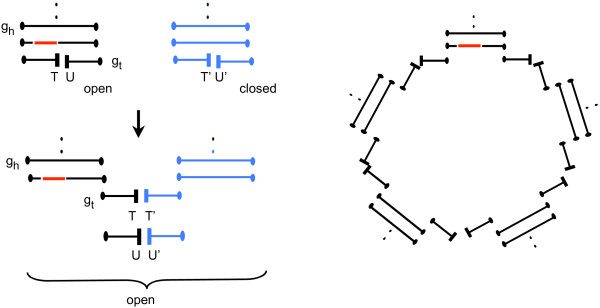
**(left) Combining an open bundle (in black) and a closed bundle (in blue) by exchanging half paths**. Dots represent free ends, rectangular blocks represent *T *vertices in half paths. Cuttable edge in red. This may be iterated to incorporate more closed bundles in a linear or circular structure as in the large open bundle on the right. Cuttable edge is in original open bundle.

Although the missing genes represented by the free ends in an open bundle will eventually be inserted in an optimal way by manipulating cuttable edges, this is not possible within closed bundles. **fillScaffolds **thus calls the second algorithm **combineBundles**, which subsumes all closed bundles within open ones, as in Figure 3, thus creating larger open bundles, including some which contain more than two telomeres. This is done in such a way as to minimize the eventual genomic distance between *G*_1 _and . This step requires interchanging the half paths of the pseudopaths in the two bundles being combined, through changes in telomere adjacencies, to maximize the number of good paths according to the Tesler formulation in Equation (2).

Finally, **fillScaffolds **calls **completeBundle**, which makes the connections between the free ends and the cuttable edges within each of the open bundles.

The output of the algorithm includes cycles, each containing at most one pair of "adjacent" telomeres, which become the two endpoints of a complete path within the breakpoint graph.

After presenting the algorithm, we state and prove a theorem establishing its correctness:

### Algorithm fillScaffolds

**Input**: A fully sequenced and assembled (without gaps) genome *G*_1_, and a genome *G*_2 _made up of scaffolds containing some of the genes in *G*_1 _and gaps.

**Output**: A completed form of *G*_2_, denoted  where the missing genes from *G*_1 _are inserted into the gaps in such a way as to minimize , and the associated breakpoint graph.

1. Construct the breakpoint graph based on genome *G*_1 _(blue edges) and *G*_2 _(red edges), including *cuttable *red edges between consecutive genes in *G*_2 _scaffolds separated by a gap. We include *T*_1 _vertices at the telomeres of *G*_1 _chromosomes and *T*_2 _vertices at the end of *G*_2 _scaffolds. We do not complete the third step of Figure 2, so the graph may contain cycles, complete paths and other paths.

2. We construct the initial bundles as follows. We choose any free end not already in any bundle as the seed of a new bundle. Then if a path containing free end *g*_*t *_is in a bundle *B*, then we also include the path with *g*_*h *_as a free end, and vice versa.

3. There can be zero or two *T *vertices in an initial bundle. If there are two, we consider the two half paths as if they were one path where the two *T *are adjacent, even though there is no red or blue edge connecting them.

4. We use **combineBundles **to remove all the closed bundles by merging them with open bundles, or with complete paths or cycles with cuttable edges, resulting in larger open bundles. We do this in such a way as to minimize .

5. Complete each bundle, using **completeBundle**.

### Algorithm combineBundles

**Input**: The set of open and closed bundles as well as the set *S *of complete paths and cycles with cuttable edges.

**Output**: A set of open bundles, and a subset *S' *of the complete paths and cycles. The open bundles contain all the vertices in the input bundles plus those vertices in *S*\*S'*, the paths and cycles not included in *S'*.

**Figure 4:**
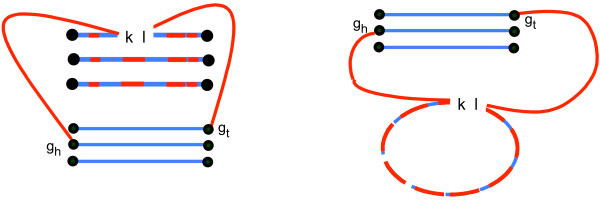
**Combining a closed bundle, represented by blue incomplete paths, with an open bundle (left) and with a cycle (right) with cuttable edge *kl*, shown here after being replaced by two cuttable edges *kg*_*h *_and *g*_*t*_*l***.

1. **while **there is a closed bundle with a *T*_1_*T*_1 _adjacency and a open bundle, or complete path with a cuttable edge, with a *T*_2_*T*_2 _adjacency, combine them by switching the adjacencies between *T *vertices, i.e., by exchanging two half-paths. This results in a larger open bundle and also increases the number of good complete paths by one.

2. **while **there is a closed bundle with a *T*_2_*T*_2 _adjacency and a open bundle, or complete path with a cuttable edge, with a *T*_1_*T*_1 _adjacency, combine them by switching adjacencies. This results in a larger open bundle and also increases the number of good complete paths by one.

3. **while **there is a closed bundle with a *T*_2_*T*_2 _adjacency and closed bundle with a *T*_1_*T*_1 _adjacency, combine them by switching adjacencies. This results in a larger *closed *bundle and increases the number of good complete paths by one. The closed bundle eventually has to be combined with an open bundle or cycle or complete path.

4. **while **there is a closed bundle with a *TT *adjacency and a open one with a *TT *adjacency, combine them by switching adjacencies. To maintain the number of good paths, if the adjacencies are *T*_1_*T*_2 _, and , then after the switching the adjacencies they should be  and .

5. **while **there is a closed bundle, combine it with an open bundle or cycle or complete path by adding a pair of cuttable edges, as in Figure 4:

i. Find two free ends *g*_*h *_and *g*_*t *_in the closed bundle.

ii. Choose a cuttable edge *kl *in some open bundle, or path or cycle.

iii. Replace *kl *by two cuttable edges *kg*_*h *_and and *g*_*t*_*l*.

### Algorithm completeBundle

**Input**: a good bundle.

**Output**: a number of cycles.

**Figure 5:**
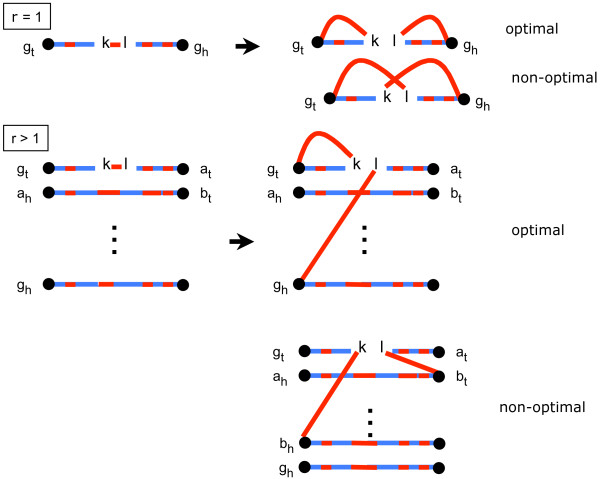
**First step in completing a good bundle, maximizing the number of cycles**. *r *is the number of incomplete paths in the bundle. Dots represent free ends before the step. *kl *is a cuttable edge in the input bundle, as are *kg*_*t *_and *g*_*h*_*l *in the new bundle. Output consists of two cycles (for *r *= 1) or one cycle and an open bundle with one fewer incomplete path (for *r *> 1).

**while **there remain paths in the bundle as in Figure 5

1. Choose a path containing a cuttable edge *kl*, with endpoint *g*_*t*_, where *l *is not on the subpath between *k *and *g*_*t*_.

2. Find the path with endpoint *g*_*h*_, possibly the same path.

3. Replace *kl *by *kg*_*t *_and *g*_*h*_*l*, which are red cuttable edges. This results in a cycle containing *kg*_*t *_and a path containing *g*_*h*_*l*, unless *g*_*t *_and *g*_*h *_are on the same path, in which case the operation produces two cycles.

#### Proving the algorithm

After the first three steps of **fillScaffolds**, suppose we have constructed *γ *open bundles with r_1_, ⋯, r_*γ *_paths, *β *closed bundles not containing *T *vertices with q_1_, ⋯, q_*β *_paths, and *δ *- *β *closed bundles containing *T *vertices with *q*_*β *+ 1_, ⋯, *q*_*δ *_paths. Let ϵ = 0 unless *δ *- *β *> 0 but there are no open bundles containing *T *vertices nor any complete paths with cuttable edges, in which case ϵ = 1. Suppose there were κ* cycles and p* complete paths in the original breakpoint graph of *G*_1 _and *G*_2_.

**Theorem**: There are(3)

cycles and complete paths in the final breakpoint graph constructed by **fillScaffolds**. Moreover, not only is the number of cycles *κ *maximal over all ways of inserting the missing genes, but so is the number of good complete paths *π *≤ *p*. Thus the algorithm also implicitly produces the value of *D*(*G*_1_, *G*_2_).

*Proof*: We first show that in completing an open bundle with *r *paths, we obtain *r *+ 1 cycles. Later, we will show that each of these cycles has at most two *T *vertices.

Consider the case *r *= 1. Figure 5 shows that completing this bundle in the optimal way creates two cycles. It also shows that for *r *> 1, we obtain a open *r *- 1-bundle plus one cycle. Thus, completing an open bundle with *r *paths produces a total of *r *+1 cycles.

It is thus never advantageous to draw a pair of red edges between two open bundles with *r *and *s *edges, since this cannot create a cycle, only a bundle with *r *+ *s *- 1 edges. When completed this will only give *r *+ *s *cycles instead of the *r *+ *s *+ 2 if we had completed them separately.

On the other hand, to be processed toward completion, it is necessary for a each closed bundle to be combined with either an open bundle, or a cycle or a complete path with a cuttable edges, since a closed bundle has no cuttable edges by itself. The optimum ways to do this are illustrated in Figs. 3 and 4. In the former case, where both bundles have *T *vertices, switching adjacencies allows a closed bundle with *r *paths to contribute *r *paths to the open bundle, and eventually to be responsible for *r *cycles. If one of the bundles has no *T *vertices, on the other hand, the closed bundle can contribute only *r *- 1 of its *r *paths in combining with the open bundle (Figure 4).

Now the numbers of open bundles, closed bundles with *T *vertices, closed bundles without *T *are fixed at the outset, and we can also find out if there are open bundles with *T *(or complete paths with cuttable edges) or not at the initial stage. Counting the number of cycles given by each type we arrive at the first claim of the theorem. Since each combination and completion is done optimally in the algorithm, the result for *κ *is best possible. So is *π*, through the operations minimizing the number of *T*_2_*T*_2 _edges in **combineBundles**.

It remains to show that the cycles output by the algorithm have no *T *vertices, i.e., are the kind of cycles appearing in the breakpoint graph in the second to last stage of the construction of Figure 2, or exactly two adjacent *T*, i.e., are the kind of complete paths (upon dissolution of the *TT *adjacency) appearing breakpoint graphs. Otherwise, the values of *κ *and *π *that we obtain in this theorem would not be those required for Equation (2).

**Figure 6:**
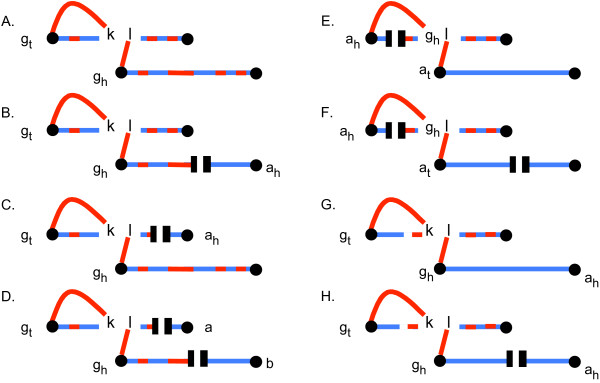
**Illustrating proof that cycles formed by **completeBundle **can contain only zero or one *TT *adjacency**. *g*_*h*_*l *is the cuttable edge formed when *kl *is destroyed as *k *is incorporated into a cycle. Case A reflects the first step in Figure 5 (*r *> 1), not involving any *TT *adjacencies. Cases B and C show how the paths from an originally closed bundle enter into the completion process thanks to the switched adjacency of a pair of half paths illustrated in Figure 3. Case D shows how two (or more) such adjacencies can accumulate in one path, but always with a cuttable edge between them. Cases E and F show how a single adjacency is incorporated in a cycle, through deletion of the cuttable edge between the adjacency and any other adjacency in the path. Cases G and H show a step in the completion of an open bundle without *T*, constructed as in Figure 4, also leading to the entry of originally closed bundle paths into the completion process.

To prove this, we refer to Figure 6, which integrates aspects of Figure 3, 4 and 5. The case by case analysis illustrated there shows that if there are more than one *TT *adjacency in a path, these adjacencies will necessarily be incorporated at most one at a time into cycles. Cycles without *TT *adjacencies are also cycles in the breakpoint graph between *G*_1 _and the augmented genome  and the cycles with *TT *adjacencies become complete paths, either good or bad, in this breakpoint graph. This completes the proof.

The construction of the optimal breakpoint graph by **fillScaffolds **inserts the missing orthologs in the scaffold gaps and at the ends of scaffolds in a way that minimizes the number of rearrangements intervening between *G*_1 _and the optimal *G*_2 _thus constructed. Once the optimal breakpoint graph is known, these rearrangements can be recovered rapidly by standard manipulations on the graph [4], as mentioned in our discussion of Equation (1). The construction of breakpoint graphs is of linear complexity, and this extends to the identification of bundles and their manipulations in **fillScaffolds**. This includes the placement of missing genes. The recovery of minimizing rearrangements can be implemented in subquadratic time [4].

### Contig fusion

The algorithm in the preceding section fills in the gaps between the scaffold whenever this is justified, so that by our definitions, the scaffolds become contigs. For unanchored scaffolds, as they are filled in by our algorithm described above, they are also being assembled into chromosomes. In doing this, our method based on the breakpoint graph treats the incorporation of each scaffold/contig as if it were a chromosomal fusion operation.

We previously found [3] through simulations that for ordinary genomes, i.e., complete gene orders, if there are *τ *rearrangements, but the genomic distance algorithm infers *D *rearrangements, then the expectation(4)

An estimate of *τ *is(5)

where *λ*_1 _and *λ*_2 _are parameters that depend on how the rearrangements are generated.

When one of the genomes consists of unanchored contigs (or filled-in scaffolds), we have to correct the output of the genomic distance algorithm *D*_*S *_before using (5) to take into account the number of "fusions" necessary to optimally piece together the contigs into chromosomes. The corrected distance is(6)

where *α*(*τ*) is a decreasing function of the number of rearrangements *τ*, approximately paralleling the derivative of *D*, namely .

### Missing genes: absent or just unsequenced?

We will use *G*_1 _and *G*_2 _here to refer to the genomes that are the source of the gene order data. By definition in our method, unsequenced genes must be located in gaps between the contigs or at the ends of scaffolds. We assume any genes within contigs have been identified. However, many or even most genes that are in *G*_1 _but have no ortholog in the *G*_2 _data may actually be absent from the latter genome either because over time they have been deleted from *G*_2 _, or because they were acquired by *G*_1 _but not by *G*_2 _since the two lineages diverged.

The scaffold filling algorithm is designed to enhance sequence assembly, and cannot distinguish one type of missing gene from another. Indeed, where gene models are available from cDNA or EST data, we could simply discard the missing genes from *G*_1 _that are not reflected in the set of gene models for *G*_2_. In general, however, we do not have this information, and the best we can hope for is to be able to estimate quantitatively how many of the missing genes are present in the genome, but unsequenced.

Let  represent the genome *G*_1 _with all the genes missing from *G*_2 _deleted. The remaining genes are ordered in the chromosomes in the same way as in *G*_1 _. One way to estimate the proportions of the two types of missing genes is to compare the genomic distance , where only the genes in common in the data from the two genomes are considered, with the distance  after *G*_2 _is augmented to  by the scaffold-filling procedure. As detailed below, we have found in extensive simulations that if all unsequenced genes were originally located in regions that are gaps after the (partial) sequencing and assembly are finished, the distances  and  are identical, or almost so, over a wide range of genome sizes, rearrangement distances and missing gene sets. If on the other hand, many of the missing genes are in reality absent from the *G*_2 _genome, a major proportion of these, approximately equal to the coverage of the genome sequencing, will have been in syntenic contexts in *G*_1 _that are in contigs in *G*_2_. Thus forcing these genes to be in gaps, as the scaffold-filling algorithm does, will tend to increase the rearrangement distance . Then if *m *is the number of missing genes(7)

is a measure of how the proportion of missing genes are not actually in the *G*_2 _genome.

The value of *D' *depends on how much the contigs are already rearranged in the independent evolution of the two genomes. If the contigs are highly rearranged compared to *G*_1 _, then there is no necessary increase in *D' *when the missing gene is forced into a gap. But if the syntenic context of a missing gene is intact in a contig, then forcing this gene into a gap remote from this context will necessarily increase *D'*.

Our strategy for evaluating this dependence requires us to manipulate the overall degree of syntenic context conservation while keeping *D *fixed. In the simulations in the **Results **section below, we accomplish this by using fixed length inversions. By generating the genomic divergence with very short inversions, we require more inversions to attain the same inferred *D*, but we also guarantee the existence of a good number of conserved segments (conserved syntenic contexts) and allow *D' *to increase. By fixing the inversion length at successively higher values, the scope of each inversion becomes longer and it is less likely a conserved segment will remain undisrupted, and *D' *will tend not to increase.

## Results and discussion

In this section we apply the scaffold filling and contig fusion methods by comparing the draft genome of *Ricinus communis *with the more complete genome of *Vitis vinifera*. We will do this in three stages. First we will give a brief description of the phylogenetic relationship of these two angiosperms and a preliminary bioinformatic comparison of their genome sequences. This will give us 14,033 presumptive orthologous genes in the two genomes, plus 4267 genes *Vitis *genes which are not in the *Ricinus *data, either because they are in the unsequenced parts of the genome, or because they have simply been deleted from, or never acquired in, the *Ricinus *lineage. We call these *missing *genes. We calculate statistics about how the missing genes are distributed in *Vitis*, as singletons, pairs, triples or longer runs. We also calculate for *Ricinus *the distribution of the number of genes per contig and per scaffold, the number of contigs per scaffold and the total numbers of contigs and scaffolds.

In the second stage, we use these distributions to simulate random pairs of genomes having the same characteristics as the *Ricinus-Vitis *data set. We model the number and distribution of missing genes as being due to three types of process:

• the evolutionary divergence of gene complement between the two species,

• the variability of conserved segment size as chromosome inversions disrupt gene order over time, and

• the distribution of contig and scaffold sizes produced during the sequencing project.

The simulations enable us to predict how these factors affect the results of scaffold filling.

Finally, in the third step, we apply our scaffold-filling algorithm and contig fusion analysis to the *Ricinus-Vitis *data and interpret the results in the light of missing gene models we elaborate and the simulations we carry out.

### The castor bean genome

Sequenced by the Sanger method to a depth of 4×, the castor bean genome exemplifies the kind of final product that we can increasingly expect of draft genome sequencing projects, with a large number of scaffolds (> 28,000) not anchored to any chromosome. (Indeed, later genomes sequenced with the 454 and Solexa methods will have shorter reads and have perhaps even shorter scaffolds.) Almost all of the genes, however, are found on a smaller number (≈ 1600) of the larger scaffolds (> 10 Kbp). To illustrate our method, we wish to pick a completely sequenced genome with which to compare *Ricinus*, one from a not too distantly related angiosperm species, so that it is likely to share a large majority of its gene complement and gene order with *Ricinus*. More distant relatives might also work, but divergent gene complement and decreasing synteny would lead to more ambiguous and less reliable results. Moreover, there is another, more stringent, condition. On the two lineages from their common ancestor leading to the two genomes, there should be no whole genome duplication (WGD) event. Though we know how to compare gene orders of such former tetraploids with diploids that diverged before the WGD [2,7], in the first instance we would like to avoid such complexities in testing our new procedure. This eliminates *Arabidopsis, Oriza, Populus *and *Medicago *among the high-quality genome sequences available. It also eliminates the closely related *Hevea brasiliensis *(rubber) genome, in the same family (Euphorbiaceae) as *Ricinus*, for which the draft sequence has been announced, but which is a recent tetraploid or, more accurately, an amphidiploid [8], p. 278.

Fortunately, there seems to be no WGD in the lineages leading to *Vitis vinifera *and *Ricinus *since their last common ancestor, and so we can use *Vitis *as *G*_1 _in our method and *Ricinus *as *G*_2_. Although Burleigh *et al*. [9] have suggested that there have been one or more WGD events in the rosid clade rooted after the divergence of *Vitis vinifera*, in the lineage leading the Euphorbiaceae family, which contains *Ricinus*, the evidence presented in that paper, namely a large number of gene families originating in the early period, is not at all statistically significant, may be a methodological artifact as acknowledged by the authors, and, *pace *reference [10], is uncorroborated in the literature (cf the recent survey of the many angiosperm WGD events by Soltis *et al*. [11]). In addition, though a relatively recent WGD has been proposed for *Vitis *[10], this suggestion has not met with general acceptance [11,12] either. Thus we may provisionally treat the *Vitis-Ricinus *relationship as being uninterrupted by WGD. Finally, there is evidence that *Vitis *gene order has evolved relatively slowly, e.g., Reference [2].

We extracted scaffold, contig and gene level data on *Ricinus communis *from GenBank as well as chromosomal gene order data on *Vitis vinifera*. Of the 18,300 *Vitis *genes, 14,033 showed up as best reciprocal hits (BRH), using BLASTP and a 1e-5 threshold to compare the proteins, among the 31,221 possible protein genes suggested by the *Ricinus *sequence. We discarded the rest of the *Ricinus *gene models.

Key statistics are given in Table 1. To the 4267 missing orthologs we add 339 genes that were found on *Ricinus *scaffolds with no other genes, i.e., since they contribute no gene order information, so that a total of 4606 genes are to be placed relative to the *Ricinus *gaps. The remaining 13,694 of the 14,033 *Ricinus *orthologs were organized into 748 (=1087-339) scaffolds each with two or more genes, i.e., containing at least some order information. The scaffolds also contained a total of 2527 gaps. Note that our algorithm automatically places additional gaps at the two ends of each scaffold, so that we need not worry separately about placing genes between the scaffolds.

**Table 1:** Statistics on *Ricinus assembly*.

genes per contig	number of contigs	contigs per scaffold	number of scaffolds	*Vitis *genes per deleted block	number of deletions
1	1699	1	469	1	2253
2	612	2	181	2	548
3	337	3	121	3	167
4	210	4	80	4	36
5	140	5	67	5	23
6	107	6	29	6	8
7	91	7	25	7	5
8	55	8	19	8	1
9	38	9	27	9	1
10	36	10	10	10	0
>10	289	>10	59	>10	3

total: 14033	3614	total: 3614	1087	total: 4267	3045

The distance , where only the orders of the 13,694 genes in both *G*_1 _and the scaffolds of *G*_2 _are considered, is 8283. The distance , which compares *G*_1 _to the augmented version of *G*_2_, namely , after the scaffold-filling procedure has been applied, so that the orders of all 18,300 genes are considered, is 9931.

### Simulations

We simulated pairs of genomes with number of genes *n *= 18, 300. The first, *G*_1_, simply has the genes evenly distributed among the 10 chromosomes. Blocks totalling 4267 genes, distributed as in Table 1, to be eventually deleted in forming *G*_2_, were chosen at random along the genome, constrained only from overlapping or even touching. At first these genes were only marked, but not deleted. For a range of values of *τ*, we applied *τ *random rearrangements to *G*_1 _and then deleted the marked genes. We assumed that rearrangements are preponderantly inversions (around 90%), a common tendency in gene order evolution, and we chose the two breakpoints for each rearrangement randomly along the chromosome.

#### All deletions create gaps

Each deletion event created a gap between two contigs. In addition random contig breaks were inserted to make sure that the number of contigs totaled 3614, as in the *Ricinus *data in Table 1. Adjacent contigs were then assembled randomly into scaffolds in such a way as to produce the same distribution of contigs per scaffold as in Table 1. Single-gene scaffolds were identified and removed from the lists of scaffolds and contigs and transferred to the list of missing genes, as in the *Ricinus *analysis.

Applying the **fillScaffolds **algorithm to these data, for *τ *= 3000, 6000,⋯,15000, twenty runs for each *τ*, demonstrated that under the model where missing genes are entirely due to incomplete sequencing, the distance ) was exactly the same as  in 90% of the runs, and 2 rearrangements more costly (out of 5000 or more) in the remaining cases. Thus we can conclude that **fillScaffolds **generally inserts the 4267 missing genes (plus a variable number of genes from single-gene scaffolds) at virtually no cost, in terms of genomic distance. This holds over a wide range of genomic distances. It also holds for a range of models of rearrangement; for example, if instead of the two breakpoints being randomly chosen over the chromosome, we restrict inversions to involve only a small number of genes, the difference between pre- and post-scaffold-filling is less than 0.5%.

We note that the simulations, including the use of our implementation of the **fillScaffolds **algorithm, took on the order of a minute each on a MacBook Pro with 3.06 GHz processor speed.

#### Some deletions do not create gaps

How can we model the subset of missing genes that are not those unsequenced genes in *G*_2 _that cause gaps between the contigs, but genes that are not in the *G*_2 _genome at all? To do this, we delete some proportion of the genes marked at the beginning of the simulation as before, but do not create a gaps between contigs at the deletion point. Insofar as the syntenic context of the absent *G*_1 _gene is conserved in a *G*_2 _contig, this should cause an increase in  over , due to the rearrangement cost of moving the gene from its original context to a gap. It will not tend to cause an increase if the syntenic context in *G*_1 _has already been rearranged in *G*_2_, e.g., if the absent gene is at the breakpoint of an inversion or translocation. Because this effect involves the interaction of synteny conservation and rate of non-gap-creating deletions, we set up simulations as described in the **Missing genes: absent or just unsequenced? **section above, varying both of these processes. We carried out simulations with from 60% to 100% non-gap-creating deletions and with fixed-length inversions from 1 to 6 genes long. Each of the 30 simulation conditions (6 conservation settings times 5 deletion types) is represented by the average of 20 simulation trials.

**Figure 7:**
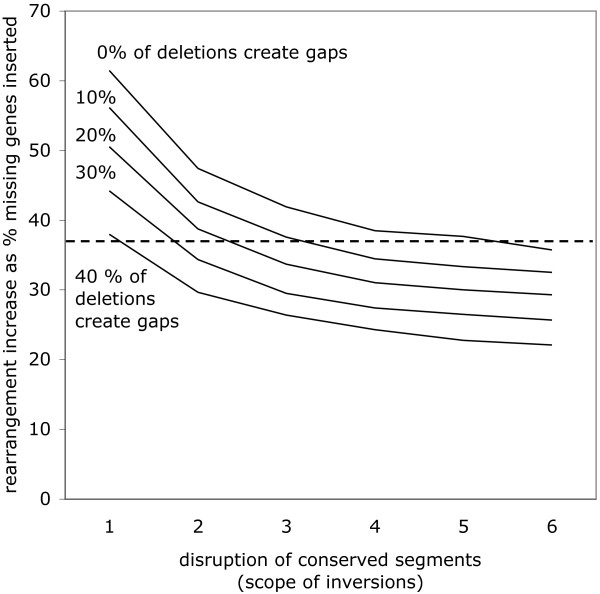
**Effect on *D' *of decreasing syntenic conservation for different proportions of non-gap-creating deletions**.

**Figure 8:**
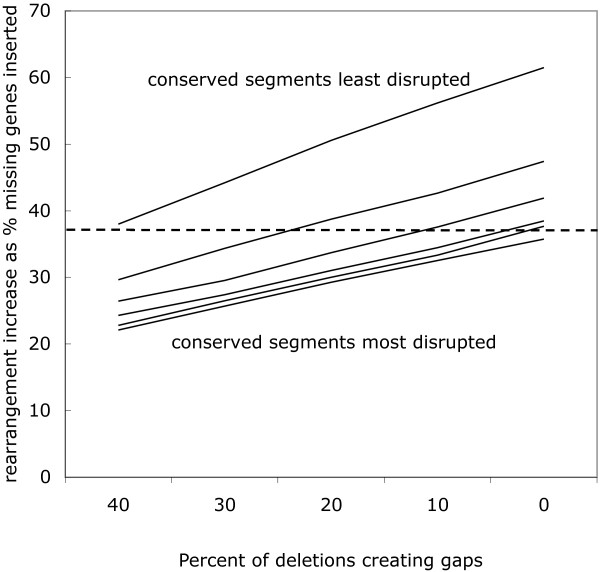
**Effect on *D' *of increasing non-gap-creating deletions for different levels of syntenic conservation**.

The simulations show that the value of *D' *increases with greater conserved synteny, and with higher proportions of non-gap-creating deletions. This is depicted in two ways in Figures 7 and 8. Of particular interest will be the case of *D' *= 0.37 indicated by the dashed line in both graphs. This case corresponds to the *Ricinus-Vitis *comparison as reported in the **Results on ***Ricinus *section below.

### Distances

**Figure 9:**
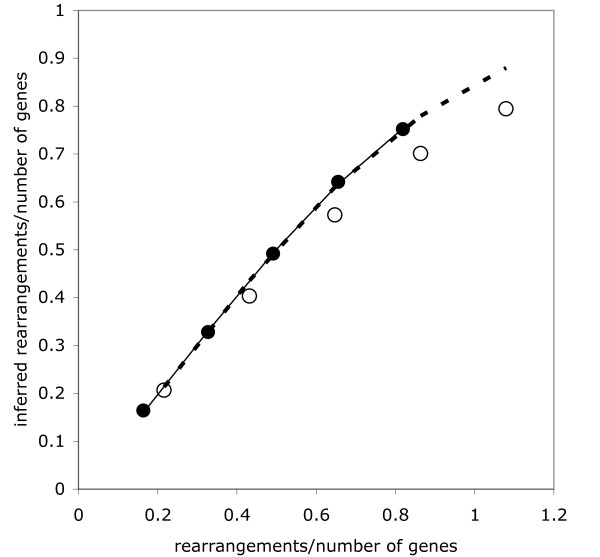
**Fit of equation (4) (solid line) to the normalized number of rearrangements inferred (filled dots), before deletion of missing genes; *λ*_1 _= 0.899, *λ*_2 _= 0.988**. Fit of same equation, when taking into account only genes remaining after deletions and scaffold construction (dashed line), to normalized number of rearrangements inferred (open dots) after correction for the number of scaffold fusions. The position of each dot is based on an average of 20 trials.

We compare the relationship between the inferred number of rearrangements, corrected for the number of scaffolds in *G*_2_, and the actual number of random rearrangements *τ *used in simulating this genome. Before the deletion of the genes from the gaps and the creation of the scaffolds, i.e., when the genomes contain 18,300 orthologs, equation (4) closely predicts the observed distances. This is illustrated in Figure 9, which is based on the average of 20 simulated trials per data point.

After the deletions of 4267 genes representing those absent from *G*_2_, as well as the variable number (usually less than 200) genes in single-gene scaffolds, following scaffold creation in the "all deletions create gaps" model, the observed distance is less than that predicted by equation (4), especially when the simulated rearrangements become numerous. This is also illustrated in Figure 9. The observed distance (averages of 20 trials) is corrected (downwards) for the 935 chromosomal fusions necessary to assemble the contigs (filled scaffolds) into 10 chromosomes, using  in equation (6), but the inferred distance is smaller than predicted even without this correction.

These results indicate that estimating *τ *using equation (5), e.g., for the purposes of distance-based phylogeny, is likely to underestimate this genomic distance to some extent.

### Results on *Ricinus*

Our algorithm found a distance of 9931 operations between *Vitis *and the reconstructed *Ricinus *genome, corrected for fusions to 9365 by subtracting 566 fusion operations.

In previous work [7] we estimated the distance between *Populus *and *Vitis*, which should represent the same divergence time, given that *Populus *and *Ricinus *presumably shared a common ancestor since the divergence of the *Vitis *lineage. We also estimated [2] the distance between *Populus *and *Carica papaya*, which should represent a divergence time smaller than *Vitis - Ricinus*. Making these comparisons (Table 2) is reasonable, although the *Populus *rearrangements occurred after a WGD event.

**Table 2:** Normalized distances and insertion costs for three comparisons.

	without missing homologs			missing homologs replaced			change		
Comparison	*n*	*D*	*D*/*n*	*n*	*D*	*D*/*n*	Δ*n*	Δ *D*	
*Populus*^*b *^-Vitis [7]	2104	1092	0.52	6144	2545	0.41	4040	1453	0.36
*Populus*^*b *^-Carica [2]	2590	1461	0.56	7222	3466	0.48	4632	2005	0.43
*Ricinus*^*c*^-Vitis	13694	8283	0.60	18300	9931	0.54	4606	1682	0.37
*R.-V. corrected*^*a*^	13694	7715	0.56	18300	9365	0.51	4606	1684	0.37

*a*: contains corrections to distance due to scaffold fusions. *b: Populus *comparisons include rearrangements during the re-diploidization of the ancestral tetraploid. *c*: some missing homologs in the *Ricinus *comparisons are simply not sequenced, while in the other comparisons, missing homologs are virtually all absent from the genome.

When all the data are taken into account, and each distance normalized by the number of genes in the comparison, the *Vitis - Ricinus *distance is comparable to the *Populus - Carica *one, and both are greater than *Populus - Vitis*. This slight disproportion between *Vitis - Ricinus *and *Vitis - Populus *is attributable, in unknown proportions, to

• the use of a method more refined than BRH, namely OrthoMCL [13], to identify *Populus - Vitis *orthologs. For *Vitis - Ricinus *we used BRH without any validation by chromosomal context or by gene ontology.

• generation time difference in different lineages, as argued in [2].

• the proportion of non-gap-creating deletions, which is a function of the divergence in gene complement.

Only the first of these is directly amenable to computational improvements, without further biological input.

The key result in Table 2 is the rate of correct placement of the missing orthologs. Some 63% percent of the orthologs were inserted without any increase in rearrangement distance. This is comparable to the 57% - 64% in the previous studies, even though the latter each benefited from evidence from two syntenic contexts rather the single *Vitis *contexts used for orthologs placement in *Ricinus*. With reference to Figures 7 and 8, it suggests that around 75% of missing genes are not attributable to incomplete sequencing, but rather to divergent gene complement in the two genomes. Table 2 and Figure 7 and 8 are also compatible with the fact that almost all the missing genes in the *Populus *comparisons are attributable to divergent gene complement.

## Conclusions

One methodological difficulty inherent in our comparison of *Ricinus *and *Vitis *is that of ortholog identification. BRH, which we used, is the simplest approach to this problem, using only sequence similarities, but there are many others available such as OrthoMCL [13], Inparanoid [14] and MSOAR [15], which can also make use of local order and gene ontology information.

Aside from improvements in orthology identification, which is a major roadblock to all gene order reconstruction problems, not only the scaffold assembly problem discussed here, there are a number of immediate possibilities to extending our technique. One is to take into account gap sizes on the scaffolds and gene sizes for the orthologs. As it is our reconstruction does not limit how many genes of whatever size can go into a gap.

A second, associated, problem would be to allow overlapping scaffolds, in cases where the paired ends data might not be resolved enough to preclude this configuration. We have already done this to some extent, in treating the single-gene scaffolds in the same way as missing genes. These small scaffolds are thus being inserted into the gaps in other scaffolds. Allowing more general overlapping might complicate the algorithm, but in practice this could be a rare occurrence.

In the present work, we have assumed *G*_1 _to be fully sequenced and *G*_2 _to be in scaffolds. This is reasonable even though there are some gaps in the *Vitis *genome; there are not likely to be a large proportion of genes that remain unsequenced as there are in *Ricinus*. In other contexts, however, it might be desirable to expand our theoretical and practical considerations to allow both genomes to be in scaffold form. Here it may be necessary to insert missing orthologs in both directions, from *G*_2 _to *G*_1 _as well as from *G*_1 _to *G*_2_.

We have devoted much effort to differentiating between unsequenced genes and genes that are truly absent from the genome. Our goal here has been to predict the location of those genes that are missing because of incomplete sequencing or unsuccessful gene identification, not those genes that are absent because they have been deleted from *Ricinus *over time or acquired by *Vitis *since the divergence of the two lineages. Yet the latter class of genes are forced into our *Ricinus *reconstruction, because we have no *a priori *way of knowing they are actually absent from *Ricinus*. Our procedure would work equally well if instead of using all the missing *Vitis *genes, we used only those for which we had unigene, EST, RNA sequence or other cDNA evidence of their existence in *Ricinus*. We could then apply our algorithm to reconstruct the *Ricinus *gene order based on that of a reduced version of *Vitis *where all the genes with no *Ricinus *ortholog would be deleted at the outset from the *Vitis *gene order.

We discussed the case of BAC sequencing where the scaffolds are anchored on chromosomes so that there is no issue of optimal scaffold fusion. Gaps can still occur between BACs, and even inside BAC sequence assemblies, depending on the strategies and policies of the sequencers. Here our algorithm would require no modification to do a rearrangement analysis and ortholog insertion.

There are many occurrences of non-uniqueness in rearrangement inference and ortholog insertion in applying methods such as ours. This precludes a straightforward comparison of  with the pre-deletion simulated genome to validate the method. However, non-uniqueness can sometimes be partially resolved by examining elements in common from many optimal solutions.

It bodes well for future use of this methodology that our algorithm was efficient enough to solve the problem with over 18,000 genes in less than a minute of computing time on a laptop computer, putting virtually all genomes within range of this technology.

## Availability

The program implementing scaffold filling is included in this paper as Additional file 1.

## Authors' contributions

AM, CZ and DS formulated the problem, devised and proved the algorithms, carried out the data analysis and simulations, and wrote the paper. QZ constructed the scaffold data base. VAA and SR provided motivation and help in formulating the problem, suggested the genomes to analyze and helped interpret the results and write the paper. All authors read and approved the final manuscript.

## Supplementary Material

Additional file 1 **Contains the Java code for the fillScaffolds algorithm, including class files and sample input data, as well as user instructions and a list of environments in which the program has been tested**. Click here for file
